# Biomarkers in Pemphigus Vulgaris: A Systematic Review

**DOI:** 10.1177/12034754241266136

**Published:** 2024-07-29

**Authors:** Ryan S. Q. Geng, Bethany Wilken, Siddhartha Sood, Ronald G. Sibbald, Cathryn Sibbald

**Affiliations:** 1 Temerty School of Medicine, University of Toronto, Toronto, ON, Canada; 2 Faculty of Medicine, Queen’s University, Kingston, ON, Canada; 3 Dalla Lana School of Public Health & Division of Dermatology, Department of Medicine, University of Toronto, Toronto, ON, Canada; 4 Division of Pediatric Dermatology, The Hospital for Sick Children, University of Toronto, Toronto, ON, Canada

**Keywords:** pemphigus vulgaris, biomarkers, immune pathways

## Abstract

**Introduction:**

Pemphigus vulgaris (PV) is a rare intraepidermal blistering disease that is potentially life-threatening due to risk of infection and failure of skin barrier function. The identification of biomarkers has the potential to provide diagnostic utility and identify new therapeutic targets. The objective of this systematic review is to identify all potentially relevant PV biomarkers, categorize them, and identify trends to determine the involvement of T-cell-mediated, B-cell-1mediated, and innate immune-mediated pathways in PV pathogenesis.

**Methods/Results:**

Medline and Embase databases were searched according to Preferred Reporting Items for Systematic Reviews and Meta-analyses guidelines, resulting in the inclusion of 66 studies that reported on a total of 2463 patients and 146 unique biomarkers. Biomarkers were categorized into T-cell-mediated, B-cell-mediated, and innate immune system pathways. The most notable biomarkers trends include elevations in IL-4, IL-6, IL-17A, anti-Dsg1/3 autoantibodies, and a reduction in T_reg_ cells and FOXP3.

**Conclusion:**

The results of this review support current theories of PV pathogenesis, with increased T_h_2 activity, increased T_h_17 activity, decreased T_reg_ activity, and production of anti-Dsg1/3 autoantibodies being observed. Targeting of IL-4 and IL-6 may provide therapeutic benefit. However, more research is required to validate biomarkers for clinical utility and assess viability as therapeutic targets.

## Introduction

Pemphigus vulgaris (PV) is a rare intraepidermal blistering disease. The mean age of onset is between 40 and 60 years, with a global incidence of 2.83 per million population.^1^ PV is characterized by flaccid blisters on a normal or erythematous base with a preference for the oral mucosa, scalp, face, axillae, trunk, and groin. Due to the fragility of the blisters, they often break, leading to painful erosions that are prone to infection.^2^ In most cases, the oral mucosa is the first area involved, and 90% of PV patients develop oral lesions throughout the course of the disease.^3^ PV is a psychosocially ladened disease, with many patients experiencing feelings of anxiety and depression.^4^ Painful oral lesions can also deter patients from eating, resulting in weight loss and nutritional deficiencies.

The development of PV centres around the production of autoantibodies targeting desmoglein (Dsg) 1 and 3, which are cadherin proteins involved in the formation of desmosomes. Targeting of Dsg1 and Dsg3 results in the breakdown of cell-cell adhesion between keratinocytes, resulting in acantholysis.^5^ Due to differential expression of Dsg proteins in skin and mucosa, anti-Dsg1 is responsible for cutaneous lesions, while anti-Dsg3 is responsible for mucosal lesions. In addition, anti-Dsg1 and anti-Dsg3 levels correlate with disease severity.^3^ The pathogenesis of PV is not well understood, but involves loss of self-tolerance in B- and T-cells. This leads to production of autoantibodies against Dsg1 and Dsg3, release of pro-inflammatory cytokines, and loss of cell-cell adhesion that ultimately results in the formation of blisters observed in PV.^5-7^ While complement deposition may occur, it is not believed to be contributory to the pathogenesis of PV.^8^

Biomarkers are indicators of normal or pathologic biological processes that can be used to measure response to external intervention, with applications including disease diagnosis and prognosis, treatment selection, and use as an objective measure of treatment efficacy in trials.^9^ The role of hemoglobin A1c in the management of type II diabetes mellitus and the use of tumour necrosis factor (TNF)-α, interleukin (IL)-12/23, and IL-17 inhibitors in treating severe recalcitrant cases of psoriasis are just a few examples of the clinical utility of biomarkers.^10,11^ Given the potential for biomarkers to improve understanding of PV pathogenesis and provide therapeutic targets, the aim of this systematic review was to identity and categorize potentially relevant PV biomarkers into different immune pathways.

## Methods

### Literature Search

The systematic review (PROSPERO CRD42023482384) was conducted according to the Preferred Reporting Items for Systematic Reviews and Meta-analyses (PRISMA) guidelines.^12^ Medline and Embase databases were searched from inception to November 2023, resulting in 745 articles for screening after removal of 280 duplicates. A PRISMA flow diagram is provided (Supplemental Figure 1). Full search strategy is provided in the Supplemental Material.

### Inclusion and Exclusion Criteria

All published articles reporting on biomarker levels in serum, blister fluid, or skin of PV patients in comparison to healthy controls were included. Animal studies were excluded.

### Data Extraction

Articles were screened by RSQG and BW on basis of title and abstract, followed by full-text screening to determine eligibility for inclusion. Potentially relevant article references were also evaluated for inclusion. Disagreements were mediated by a third author. Data were analyzed with descriptive statistics. The National Institute of Health quality assessment tool was used to assess methodological quality, which provides quality ratings of “good,” “fair,” or “poor.” Only reports with a “good” rating were included.

### Data Analysis

Data were analyzed with descriptive statistics. Weighted associations were calculated by assigning studies that reported an increase of a biomarker in PV patients versus healthy controls a value of +1, no change in biomarker levels a value of 0, and a decrease in biomarker levels a value of −1. Weighted associations for each biomarker were calculated based on patient sample size. Weighted association values >0.5 indicate most PV patients exhibit elevated levels of the biomarker compared to healthy controls, values between −0.5 and 0.5 indicate most PV do not exhibit differences in biomarker levels, and values <−0.5 indicate most PV patients exhibit lower levels of the biomarker.

## Results

A total of 66 case-control studies were included in the review, with 6 being conference abstracts that provided sufficient information for inclusion (Supplemental Figure 1). The individual studies varied in PV patient sample sizes, ranging between 5 and 280 patients, reporting on a total of 2463 patients and 146 biomarkers. Most patients were off immunosuppressive therapy for at least 1 month. All patients included were in active disease stage. The biomarkers were categorized into T-cell-mediated, B-cell-mediated, and innate immune system pathways. A table summarizing the studies included in this review is provided in as Supplemental Table 1.

### T_h_1 Biomarkers

TNF-α is a characteristic T_h_1 biomarker that was elevated in PV patients compared to healthy controls. Other T_h_1 biomarkers including IL-8 and c-x-c motif chemokine ligand (CXCL)9 were also elevated However, IL-1β, interferon (IFN)-γ, c-c motif chemokine ligand (CCL)3, CXCL10, and IL-2 were largely unchanged (Supplemental Table 2).

### T_h_2 Biomarkers

IL-4 is a characteristic T_h_2 cytokine that was found to be elevated in PV patients compared to healthy controls by all included studies. Several other T_h_2 cytokines including IL-6, IL-10, and IL-13 were also elevated in PV patients. Other studied T_h_2 biomarkers including IL-31, IL-31RA, CCL2, and CCL22 remained unchanged (Supplemental Table 3).

### T_h_17 Biomarkers

The characteristic T_h_17 cytokine, IL-17A, was found to be elevated in PV patients compared to healthy controls by all included studies. Other T_h_17-related biomarkers found to be elevated in PV patients include IL-6, IL-23, CCL20, CCR6, and retinoic acid receptor-related orphan receptor (ROR)γt. IL-1β and transforming growth factor (TGF)-β remained unchanged (Supplemental Table 4).

### T_reg_ Biomarkers

While a few T_reg_ associated biomarkers including IL-10, IL-2R and CCL17 were found to be elevated in PV patients compared to healthy controls, c-c chemokine receptor (CCR)4, T_reg_ cell count and the forkhead box protein (FOXP)3 transcription factor unique to T_reg_ cells were found to be decreased. Other biomarkers including IL-2, CCL22 and TGF-β remained unchanged (Supplemental material Table 5).

### B-Cell and Antibody Biomarkers

Tumour necrosis factor super family (TNFSF)13A and TNFSF13B are B-cell proliferation and survival factors. While TNFSF13A was elevated in PV patients compared to healthy controls, TNFSF13B and B-cell counts were unchanged (Supplemental Table 6).

With regard to antibody levels, anti-Dsg1, anti-Dsg2, and anti-Dsg3 levels were all found to be elevated in PV patients in comparison to healthy controls, although ant-Dsg2 autoantibodies are not pathogenic in PV. For anti-Dsg3, IgG isoforms (IgG1/3) were increased, while IgA and IgM were unchanged (Supplemental Table 6).

### Innate Immune System Biomarkers

While several innate immune system biomarkers were found to be elevated in PV patients, the pathogenesis of PV is rooted in an autoimmunity involving driven by aberrant adaptive immune activity. Changes in innate immune activity are likely due to influence from cytokines released by components of the adaptive immune system. Oxidative stress may also be a feature of PV pathogenesis (Supplemental Table 7).

## Discussion

This review included 66 studies, reporting on 146 unique biomarkers. The biomarker trends identified support the role of a T_h_2 skewed response, increased T_h_17 activity, reduced T_reg_ activity, and anti-Dsg1/3 autoantibodies in the pathogenesis of PV. Biomarkers identified can potentially be applied in assessing treatment efficacy, offer support for targeted therapy and clinical assessment.

**Figure 1.**
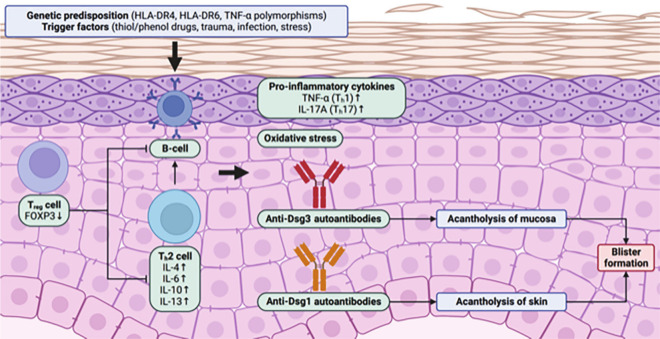
Schematic illustration of PV pathogenesis. HLA-DR4, HLA-DR6 serotypes and TNF-α polymorphisms have been associated with PV. Several trigger factors of PV have also been identified including thiol and phenol drugs, trauma, infection and stress. The reduction of T_reg_ cell activity and increased T_h_2 cell activity leads to activation of autoreactive B-cells producing autoantibodies against Dsg1 and/or Dsg3. Anti-Dsg1/3 autoantibodies are primarily of the IgG1 and IgG4 isotypes. Dsg3 is present on mucosal keratinocytes, resulting in acantholysis of mucosa. Dsg1 is present on cutaneous keratinocytes, leading to acantholysis of skin. In either case, acantholysis results in formation of flaccid blisters that break easily, leaving erosions that are characteristic of PV. Elevated levels of pro-inflammatory cytokines including TNF-α and IL-17A may also be present. However, their role in PV pathogenesis remains unclear. Created with BioRender.com. PV, pemphigus vulgaris; HLA, human leukocyte antigen; TNF, tumour necrosis factor.

The pathogenesis of PV centres around autoimmunity due to the production of anti-Dsg1/3 autoantibodies, but also involves an interplay with T-cell responses (Figure 1). PV more commonly presents in individuals with genetic predisposing factors including the human leukocyte antigen (HLA)-DR4 and HLA-DR6 serotypes.^13^ Several trigger factors for PV have been identified including phenol and thiol drugs, infection, trauma, and stress.^14^ The activation of B-cells producing anti-Dsg1/3 autoantibodies is promoted by interactions with T_h_2 cells through the action of cytokines including IL-4, which also promotes isotype switching from IgG1 to IgG4.^15^ Isotypes of anti-Dsg3 autoantibodies identified in this study were primarily IgG1 and IgG4 (Supplemental Table 6). The role of T_h_2 activity is largely supported by the results of this review, as levels of T_h_2 cytokines including IL-4, IL-6, IL-10, and IL-13 were found to be elevated in PV patients (Supplemental Table 3). In particular, IL-6 has been found to correlate with PV disease severity.^16^ T_reg_ cells have potent immunosuppressive functions through inhibiting T-cell activation, differentiation, and effector functions including T-cell cytokine production and B-cell antibody production.^17^ The increased T_h_2 activity and loss of self-tolerance suggests reduced T_reg_ activity, which is supported by the results of this review as levels of FOXP3, a transcription factor specific to T_reg_ cells, is consistently reduced in PV patients (Supplemental Table 5). T_h_1 cell responses are believed to play a minor contributory role to PV pathogenesis, as genetic polymorphisms in TNF-α have been found to be associated with PV and levels of TNF-α correlate with PV disease severity.^16,18^ However, the mechanism remains unclear. T_h_17 cell response appears to be elevated in PV patients, as its characteristic IL-17A cytokine was consistently elevated (Supplemental Table 4). However, it is unclear if the involvement of T_h_17 is contributory to PV pathogenesis or merely a consequence of PV, as levels of IL-17A were not found to correlate with PV disease severity.^19^ While increased oxidative stress was identified in this review, it is again unclear if this is contributory or a consequence of PV (Supplemental Table 7). In summary, the interplay between T-cell and B-cell responses, and the generation of autoantibodies targeting Dsg1 and/or Dsg3 results in loss of keratinocyte cell-cell adhesion, leading to acantholysis and formation of flaccid blisters and erosions that characterize PV.

Current PV therapy centres around the use of corticosteroids that may be combined with several adjunctive therapies including azathioprine, mycophenolate mofetil, and cyclophosphamide.^20^ While effective, these agents confer the risk of broad immunosuppression. Furthermore, some PV patients are resistant to therapy with these more conventional agents. Thus, it is important to identify biomarkers for targeted therapy. Currently, B-cell inhibitors including rituximab and TNF-α inhibitors including etanercept, infliximab, sulfasalazine, and pentoxifylline have demonstrated efficacy in treating PV.^21-23^ Given the mechanistic role of IL-4 in PV pathogenesis and the correlation of IL-6 levels with PV disease severity, it would be interesting to determine if IL-4 or IL-6 inhibitors provide therapeutic benefit in PV. Currently, there are only a few case reports assessing the use of dupilumab, an IL-4/13 inhibitor, in treating PV. Of the 4 patients reported, 3 achieved remission, while 1 experienced no improvement.^24-26^ Larger trials are required to assess efficacy of dupilumab in treating PV more definitively.

Limitations of our study include small sample sizes in individual studies, use of different laboratory techniques in assessing the different biomarkers and a heterogenous patient population. Further research is needed to validate biomarkers to provide diagnostic utility and assess viability as therapeutic targets.

## Conclusion

PV is a rare intraepidermal blistering disease that can potentially be life-threatening due to the risk of infection and loss of skin barrier function. This systematic review highlights the utility of identifying biomarkers to provide insight in PV pathogenesis and identifying potential therapeutic targets. The results of this review largely supports current theories of PV pathogenesis, identifying increased T_h_2 activity, increased T_h_17 activity, reduced T_reg_ activity, and production of anti-Dsg1/3 autoantibodies as being potentially contributory to PV pathogenesis. The most notable biomarkers trends include elevations in IL-4, IL-6, IL-17A, anti-Dsg1/3 autoantibodies, and a reduction in T_reg_ cells and FOXP3.

## Supplemental Material

sj-docx-1-cms-10.1177_12034754241266136 – Supplemental material for Biomarkers in Pemphigus Vulgaris: A Systematic Review Supplemental material, sj-docx-1-cms-10.1177_12034754241266136 for Biomarkers in Pemphigus Vulgaris: A Systematic Review by Ryan S. Q. Geng, Bethany Wilken, Siddhartha Sood, Ronald G. Sibbald and Cathryn Sibbald in Journal of Cutaneous Medicine and Surgery
