# Supplementary material for: Biomarkers in Pemphigus Vulgaris: A Systematic Review
Source: J Cutan Med Surg. 2024 Jul 29;28(5):458–62. doi: 10.1177/12034754241266136 (PMC11528842; doi:10.1177/12034754241266136)
Supplement: sj-docx-1-cms-10.1177_12034754241266136 – Supplemental material for Biomarkers in Pemphigus Vulgaris: A Systematic Review [file sj-docx-1-cms-10.1177_12034754241266136.docx]

**Supplementary Material**

**Search strategy**

Medline and Embase

1 "pemphigus vulgaris".ab,ti.

2 biomarker.ab,ti.

3 lymphocyte.ab,ti.

4 "T-cell".ab,ti.

5 "B-cell".ab,ti.

6 neutrophil.ab,ti.

7 "mast cell".ab,ti.

8 "dendritic cell".ab,ti.

9 "plasma cell".ab,ti.

10 macrophage.ab,ti.

11 eosinophil.ab,ti.

12 basophil.ab,ti.

13 cytokine.ab,ti.

14 chemokine.ab,ti.

15 "c reactive protein".ab,ti.

16 CRP.ab,ti.

17 erythrocyte sedimentation rate.ab,ti.

18 ESR.ab,ti.

19 complement.ab,ti.

20 "antibod* ".ab,ti.

21 (level or expression or amount or threshold or fold or titre or regulation or modulation or variation or dynamics).mp. [mp=ti, ab, hw, tn, ot, dm, mf, dv, kf, fx, dq, bt, nm, ox, px, rx, ui, sy, ux, mx]

22 desmoglein.ab,ti.

23 2 or 3 or 4 or 5 or 6 or 7 or 8 or 9 or 10 or 11 or 12 or 13 or 14 or 15 or 16 or 17 or 18 or 19 or 20 or 22

24 1 and 21 and 23

**Identification of studies via databases and registers**

**Identification**

**Screening**

**Included**

**1025** studies identified from:

Medline

Embase

Studies removed before screening:

**280** duplicates removed

**745** studies screened on basis of title and abstract

**84** studies assessed for eligibility on basis of full text

**18** studies excluded:

**9** No comparison to healthy controls

**7** No access to full text

**2** Conference abstracts with insufficient information

**66** studies included

**Figure 1.** PRISMA flow diagram.

**Supplementary Table 1.** PV biomarkers reported in the studies included in this review.

| **Article** | **N patients (mean age)** | **N controls (mean age)** | **Biomarker name** | **Biomarker type, medium** | **Value in PV patients (Mean ± SD)** | **Value in controls (Mean ± SD)** | **Overall association** | **Duration off immunosuppressive therapy** |
| --- | --- | --- | --- | --- | --- | --- | --- | --- |
| Abida 2012^1^ | 36 (45) | 78 (38) | Catalase activity | Protein, Serum | NR | NR | Increase | Untreated |
|  | 36 (45) | 78 (38) | Free thiol | Molecule, Serum | NR | NR | No change |  |
|  | 36 (45) | 78 (38) | Malondialdehyde | Molecule, Serum | NR | NR | Increase |  |
| Alecu 2009^2^ | 16 (NR) | 10 (NR) | B-cell | Cell, Serum | 15 ± 5 | 16 ± 5 | No change | >6 months |
|  | 16 (NR) | 10 (NR) | Natural killer cells | Cell, Serum | 12 ± 11 | 10 ± 7 | No change |  |
|  | 16 (NR) | 10 (NR) | T-CD3+ cells | Cell, Serum | 71 ± 12 | 73 ± 9 | No change |  |
|  | 16 (NR) | 10 (NR) | T-CD4+ cells | Cell, Serum | 48 ± 8 | 52 ± 9 | No change |  |
|  | 16 (NR) | 10 (NR) | T-CD4+/T-CD8+ cells ratio | Ratio, Serum | 2.5 ± 1.3 | 2.3 ± 0.4 | No change |  |
|  | 16 (NR) | 10 (NR) | T-CD8+ cells | Cell, Serum | 24 ± 11 | 23 ± 1 | No change |  |
|  | 16 (NR) | 10 (NR) | Treg cells | Cell, Serum | 0.022 | 0.047 | No change |  |
| Ali 2016^3^ | 23 (51) | 17 (58.9) | anti-Dsg3 IgA | Protein, Serum | NR | NR | Increase | On treatment for mean 5.4 years |
|  | 23 (51) | 17 (58.9) | anti-Dsg3 IgG | Protein, Saliva | NR | NR | Increase |  |
|  | 23 (51) | 17 (58.9) | anti-Dsg3 IgG | Protein, Serum | NR | NR | Increase |  |
| Ameglio 1997^4^ | 9 (56) | 20 (NR) | E-selectin | Protein, Serum | 45 | 28.5 | Increase | Untreated or relapsed |
|  | 9 (56) | 20 (NR) | ICAM-1 | Protein, Serum | 194 | 203 | No change |  |
| Ansari 2023^5^ | 60 (41.0) | 30 (39.1) | anti-Dsg3 | Protein, Serum | 131.5 | 2.85 | Increase | NR |
| Asashima 2006^6^ | 21 (59.5) | 22 (69.9) | BAFF | Protein, Serum | 5.01 ± 1.95 | 4.44 ± 0.77 | No change | Untreated |
| Asothai 2015^7^ | 30 (NR) | 20 (NR) | CCL2 | mRNA, Serum | NR | NR | No change | >3 months |
|  | 30 (NR) | 20 (NR) | CCL2 | mRNA, Skin | NR | NR | Decrease |  |
|  | 30 (NR) | 20 (NR) | CCL20 | Protein, Serum | 33.3 | 29.03 | Increase |  |
|  | 30 (NR) | 20 (NR) | CCL20 | mRNA, Serum | NR | NR | Increase |  |
|  | 30 (NR) | 20 (NR) | CCL20 | mRNA, Skin | NR | NR | Increase |  |
|  | 30 (NR) | 20 (NR) | CCL22 | Protein, Serum | 2259 | 1418 | Decrease |  |
|  | 30 (NR) | 20 (NR) | CCR4 | mRNA, Serum | NR | NR | Decrease |  |
|  | 30 (NR) | 20 (NR) | CCR4 | mRNA, Skin | NR | NR | Decrease |  |
|  | 30 (NR) | 20 (NR) | CCR6 | mRNA, Serum | NR | NR | Increase |  |
|  | 30 (NR) | 20 (NR) | CCR6 | mRNA, Skin | NR | NR | Increase |  |
|  | 30 (NR) | 20 (NR) | FOXP3 | mRNA, Serum | NR | NR | Decrease |  |
|  | 30 (NR) | 20 (NR) | FOXP3 | mRNA, Skin | NR | NR | Decrease |  |
|  | 30 (NR) | 20 (NR) | IL-17A | Protein, Serum | 33.61 | 26.04 | Increase |  |
|  | 30 (NR) | 20 (NR) | RORγt | mRNA, Serum | NR | NR | Increase |  |
|  | 30 (NR) | 20 (NR) | RORγt | mRNA, Skin | NR | NR | Increase |  |
|  | 30 (NR) | 20 (NR) | TGF-β | Protein, Serum | 30072 | 25272 | Increase |  |
|  | 30 (NR) | 20 (NR) | Treg cells | Cell, Serum | NR | NR | Decrease |  |
| Baroni 2012^8^ | 20 (51) | 13 (NR) | OPN | Protein, Serum | NR | NR | Increase | NR |
| Bhol 2000^9^ | 32 (NR) | 24 (NR) | IL-10 | Protein, Serum | 50.8 ± 15.04 | 1.41 ± 1.47 | Increase | Untreated |
| Chen 2018^10^ | 19 (NR) | 19 (NR) | IL-13 | Protein, Serum | 43.67 ± 3.62 | 31.44 ± 2.75 | Increase | Untreated (7), on treatment (12) |
|  | 19 (NR) | 19 (NR) | IL-4 | Protein, Serum | 28.65 ± 0.80 | 24.15 ± 1.06 | Increase |  |
| Christie 2022^11^ | 12 (NR) | 15 (NR) | CD4+ cells | Cell, Serum | NR | NR | Increase | NR |
|  | 12 (NR) | 15 (NR) | Treg cells | Cell, Serum | NR | NR | Decrease |  |
| Czech 1993^12^ | 9 (60) | 12 (35) | ECP | Protein, Blister Fluid | 435 | 3 | No change | Untreated |
|  | 9 (60) | 12 (35) | ECP | Protein, Serum | 15 | 9 | No change |  |
|  | 9 (60) | 12 (35) | IgE | Protein, Serum | 24 | 17.4 | No change |  |
|  | 9 (60) | 12 (35) | MPO | Protein, Blister Fluid | 2675 | 125 | No change |  |
|  | 9 (60) | 12 (35) | MPO | Protein, Serum | 442 | 350 | No change |  |
| Daneshvar 2023^13^ | 50 (49.2) | 56 (47.5) | BAFF | Protein, Serum | 914.4 ± 138 | 450.7 ± 48.2 | Increase | Untreated |
| Das 2018^14^ | 30 (NR) | 30 (NR) | CD163 | mRNA, Serum | NR | NR | No change | >3 months |
|  | 30 (NR) | 30 (NR) | CD163 | mRNA, Skin | NR | NR | Increase |  |
|  | 30 (NR) | 30 (NR) | CD36 | mRNA, Serum | NR | NR | Increase |  |
|  | 30 (NR) | 30 (NR) | CD36 | mRNA, Skin | NR | NR | Increase |  |
|  | 30 (NR) | 30 (NR) | IFN-γ | Protein, Serum | NR | NR | Increase |  |
|  | 30 (NR) | 30 (NR) | IL-4 | Protein, Serum | NR | NR | Increase |  |
| Das 2020^15^ | 30 (NR) | 30 (NR) | anti-Dsg1 | Protein, Serum | 72.55 ± 18.1 | 16.5 ± 1.5 | Increase | >3 months |
|  | 30 (NR) | 30 (NR) | anti-Dsg3 | Protein, Serum | 164.2 ± 40.1 | 18.2 ± 1 | Increase |  |
|  | 30 (NR) | 30 (NR) | CD40 | mRNA, Serum | NR | NR | Increase |  |
|  | 30 (NR) | 30 (NR) | CD40 | mRNA, Skin | NR | NR | Increase |  |
|  | 30 (NR) | 30 (NR) | CD80 | mRNA, Serum | NR | NR | Increase |  |
|  | 30 (NR) | 30 (NR) | CD80 | mRNA, Skin | NR | NR | Increase |  |
|  | 30 (NR) | 30 (NR) | LILRB4 | mRNA, Serum | NR | NR | Increase |  |
|  | 30 (NR) | 30 (NR) | LILRB4 | mRNA, Skin | NR | NR | No change |  |
|  | 30 (NR) | 30 (NR) | PSGL1 | mRNA, Serum | NR | NR | No change |  |
|  | 30 (NR) | 30 (NR) | PSGL1 | mRNA, Skin | NR | NR | No change |  |
| Das 2023^16^ | 30 (NR) | 30 (NR) | anti-Dsg1 | Protein, Serum | 73.7 ± 19.1 | 17 ± 1.5 | Increase | >3 months |
|  | 30 (NR) | 30 (NR) | anti-Dsg3 | Protein, Serum | 163.3 ± 40.2 | 18 ± 1 | Increase |  |
|  | 30 (NR) | 30 (NR) | CCL20 | mRNA, Skin | NR | NR | Increase |  |
|  | 30 (NR) | 30 (NR) | CCL22+ cells | Cell, Skin | NR | NR | Increase |  |
|  | 30 (NR) | 30 (NR) | CCR6+ cells | Cell, Skin | NR | NR | Increase |  |
|  | 30 (NR) | 30 (NR) | f γδTCR+ cells | Cell, Skin | NR | NR | Increase |  |
|  | 30 (NR) | 30 (NR) | FOXP3 | mRNA, Skin | NR | NR | Decrease |  |
|  | 30 (NR) | 30 (NR) | FOXP3+ cells | Cell, Skin | NR | NR | Decrease |  |
|  | 30 (NR) | 30 (NR) | IFN-γ | Protein, Serum | 75.2 ± 16.4 | 43.0 ± 10.2 | Increase |  |
|  | 30 (NR) | 30 (NR) | IL-17A | Protein, Serum | 42.0 ± 10.8 | 26.8 ± 6.3 | Increase |  |
|  | 30 (NR) | 30 (NR) | IL17A+ cells | Cell, Skin | NR | NR | Increase |  |
|  | 30 (NR) | 30 (NR) | IL-23 | Protein, Serum | 60.5 ± 12.8 | 40.3 ± 9.8 | Increase |  |
|  | 30 (NR) | 30 (NR) | IL-4 | Protein, Serum | 11.3 ± 1.9 | 9.34 ± 2.28 | Increase |  |
|  | 30 (NR) | 30 (NR) | RORγt | mRNA, Skin | NR | NR | Increase |  |
|  | 30 (NR) | 30 (NR) | TGF-β | Protein, Serum | 22.27 ± 2.44 | 25.19 ± 3.45 | Decrease |  |
|  | 30 (NR) | 30 (NR) | TGF-β+ cells | Cell, Skin | NR | NR | Decrease |  |
| D’auria 1997^17^ | 25 (NR) | 20 (NR) | TNF-α | Protein, Serum | NR | NR | Increase | NR |
|  | 25 (NR) | 20 (NR) | IL-6 | Protein, Serum | NR | NR | Increase |  |
| Deng 2020^18^ | 18 (45.8) | 14 (44.5) | Betaproteobacteria | Bacteria, feces | NR | NR | Decrease | NR |
|  | 18 (45.8) | 14 (44.5) | Burkholderiales | Bacteria, feces | NR | NR | Increase |  |
|  | 18 (45.8) | 14 (44.5) | C5a | Protein, Serum | NR | NR | Increase |  |
|  | 18 (45.8) | 14 (44.5) | Carnobacteriaceae | Bacteria, feces | NR | NR | Increase |  |
|  | 18 (45.7) | 14 (44.5) | Coprococcus | Bacteria, feces | NR | NR | Decrease |  |
|  | 18 (45.7) | 14 (44.5) | Enterobacteriaceae | Bacteria, feces | NR | NR | Increase |  |
|  | 18 (45.7) | 14 (44.5) | Enterobacteriales | Bacteria, feces | NR | NR | Decrease |  |
|  | 18 (45.7) | 14 (44.5) | Flavonifractor | Bacteria, feces | NR | NR | Increase |  |
|  | 18 (45.7) | 14 (44.5) | Gammaproteobacteria | Bacteria, feces | NR | NR | Increase |  |
|  | 18 (45.7) | 14 (44.5) | Granulicatella | Bacteria, feces | NR | NR | Increase |  |
|  | 18 (45.7) | 14 (44.5) | gut microbiota diversity | Bacteria, feces | NR | NR | No change |  |
|  | 18 (45.7) | 14 (44.5) | IL-1**β** | Protein, Serum | NR | NR | Increase |  |
|  | 18 (45.7) | 14 (44.5) | IL-2R | Protein, Serum | NR | NR | Increase |  |
|  | 18 (45.7) | 14 (44.5) | IL-7 | Protein, Serum | NR | NR | Increase |  |
|  | 18 (45.7) | 14 (44.5) | IL-8 | Protein, Serum | NR | NR | Increase |  |
|  | 18 (45.7) | 14 (44.5) | Lachnospiracea incertae sedis | Bacteria, feces | NR | NR | Decrease |  |
|  | 18 (45.7) | 14 (44.6) | YKL-40 | Protein, Serum | NR | NR | Increase |  |
| Dhandha 2012^19^ | 92 (54.7) | 47 (48.6) | anti-Dsg3 IgG3 | Protein, Serum | NR | NR | No change | NR |
|  | 92 (54.7) | 47 (48.6) | anti-Dsg3 IgA | Protein, Serum | NR | NR | No change |  |
|  | 92 (54.7) | 47 (48.6) | anti-Dsg3 IgG1 | Protein, Serum | NR | NR | Increase |  |
|  | 92 (54.7) | 47 (48.6) | anti-Dsg3 IgG2 | Protein, Serum | NR | NR | Decrease |  |
|  | 92 (54.7) | 47 (48.6) | anti-Dsg3 IgM | Protein, Serum | NR | NR | No change |  |
|  | 92 (54.7) | 47 (48.6) | anti-Dsg3 IgG4 | Protein, Serum | NR | NR | Increase |  |
| Echigo 2006^20^ | 19 (54) | 20 (55) | CCL22 | Protein, Serum | 244 ± 310 | 155.8 ± 61.0 | Increase | Untreated |
|  | 19 (54) | 20 (55) | MIG | Protein, Serum | 9.9 ± 10.1 | 3.7 ± 2.6 | Increase |  |
|  | 19 (54) | 20 (55) | CCL17 | Protein, Serum | 246.7 ± 300 | 93.3 ± 25.3 | Increase |  |
| El-Eriny 2018^21^ | 20 (39) | 10 (36) | TNFSF13 | Protein, Serum | 0.96 ± 0.80 | 0.49 ± 0.08 | Increase | Untreated |
| El-Komy 2014^22^ | 34 (39.2) | 20 (30.7) | 25OHD | Protein, Serum | 74.2 ± 53.1 | 89.7 ± 29.5 | Decrease | On treatment |
| Ergun 2023^23^ | 11 (59.9) | 7 (NR) | IL-31 | Protein, Skin | 0.063 | 0.029 | No change | Untreated |
|  | 11 (59.9) | 7 (NR) | IL-31RA | Protein, Skin | 0.167 | 0.027 | No change |  |
|  | 11 (59.9) | 7 (NR) | IL-31RA | Protein, Skin | 0.104 | 0.021 | No change |  |
| Ernst 2019^24^ | 14 (NR) | 18 (NR) | Lag-3 | Protein, Serum | NR | NR | No change | NR |
|  | 14 (NR) | 18 (NR) | Lag-3 | Protein, Skin | NR | NR | No change |  |
|  | 14 (NR) | 18 (NR) | PD-1 | Protein, Skin | NR | NR | Increase |  |
|  | 14 (NR) | 18 (NR) | PD-1 | Protein, Serum | NR | NR | No change |  |
|  | 14 (NR) | 18 (NR) | Tim-3 | Protein, Serum | NR | NR | No change |  |
|  | 14 (NR) | 18 (NR) | Tim-3 | Protein, Skin | NR | NR | Increase |  |
| Fujimura 2017^25^ | 10 (NR) | NR (NR) | CXCL5 | Protein, Serum | NR | NR | Increase | NR |
|  | 10 (NR) | NR (NR) | IL-10 | mRNA, Serum | NR | NR | Increase |  |
|  | 10 (NR) | NR (NR) | IL-36γ | mRNA, Serum | NR | NR | Increase |  |
|  | 10 (NR) | NR (NR) | CD163 | Protein, Serum | NR | NR | Increase |  |
| Funakoshi 2012^26^ | 48 (48) | 15 (55) | IgG3 | Protein, Serum | 0.058 | 0.082 | Decrease | NR |
|  | 48 (48) | 15 (55) | IgG4 | Protein, Serum | 0.033 | 0.015 | Increase |  |
|  | 48 (48) | 15 (55) | IgG | Protein, Serum | 1320 | 2800 | Decrease |  |
| Gunther 2009^27^ | 11 (NR) | 12 (NR) | CCL18 | Protein, Serum | 22 ± 1.7 | 19 ± 2 | No change | On treatment |
| Hayta 2017^28^ | 43 (51.2) | 40 (50.5) | CRP | Protein, Serum | 17.1 ± 36.5 | 4.2 ± 0.3 | Increase | Untreated |
|  | 43 (51.2) | 40 (50.5) | Hematocrit | Protein, Serum | 41.0 ± 4.8 | 44.2 ± 3.9 | Decrease |  |
|  | 43 (51.2) | 40 (50.5) | Hemoglobin | Protein, Serum | 13.7 ± 1.7 | 14.8 ± 1.5 | Decrease |  |
|  | 43 (51.2) | 40 (50.5) | MCV | Protein, Serum | 86.1 ± 9.9 | 90 ± 4.0 | Decrease |  |
|  | 43 (51.2) | 40 (50.5) | MPV | Protein, Serum | 9.2 ± 1.3 | 9.8 ± 1.2 | Decrease |  |
|  | 43 (51.2) | 40 (50.5) | N/L rate | Ratio, Serum | 2.9 ± 2.4 | 1.9 ± 1.4 | Increase |  |
|  | 43 (51.2) | 40 (50.5) | Neutrophils | Cell, Serum | 5.2 ± 2.9 | 3.7 ± 0.8 | Increase |  |
|  | 43 (51.2) | 40 (50.5) | P/L rate | Ratio, Serum | 152 ± 115 | 118.3 ± 65.8 | Increase |  |
| Joshi 2014^29^ | 30 (32) | 10 (NR) | IL-17A | Protein, Serum | 203.7 ± 105 | 0 | Increase | NR |
|  | 30 (32) | 10 (NR) | TGF-β/IL-17A ratio | Ratio, Serum | 30.3 ± 28 | 1363 ± 559.5 | Decrease |  |
|  | 30 (32) | 10 (NR) | TGF-β | Protein, Serum | 5223 | 6817 ± 2967 | No change |  |
|  | 30 (32) | 10 (NR) | Vitamin D | Molecule, Serum | 11.1 ± 5.8 | 12.1 ± 9.2 | No change |  |
| Karimi 2019^30^ | 24 (45.7) | 24 (NR) | HERV-H env | DNA, Serum | 39.3 ± 9.28 | NR | Increase | NR |
|  | 24 (45.7) | 24 (NR) | HERV-K (HML-2) env | DNA, Serum | 37.55 ± 9.2 | NR | Increase |  |
|  | 24 (45.7) | 24 (NR) | HERV-W env | DNA, Serum | 36.39 ± 8.6 | NR | Increase |  |
| Ketabi 2019^31^ | 53 (45.6) | 38 (44.2) | OPN | Protein, Serum | 11.08 ± 5.24 | 8.47 ± 5.68 | Increase | Untreated (25), on treatment (28) |
| Khil'chenko 2020^32^ | 100 (NR) | 105 (NR) | IgA | Protein, Serum | 0.28 | 0.23 | No change | NR |
|  | 100 (NR) | 105 (NR) | IgG1 | Protein, Serum | 2.29 | 1.79 | Increase |  |
|  | 100 (NR) | 105 (NR) | IgG2 | Protein, Serum | 0.91 | 1.05 | No change |  |
|  | 100 (NR) | 105 (NR) | IgG3 | Protein, Serum | 0.25 | 0.3 | Decreased |  |
|  | 100 (NR) | 105 (NR) | IgG4 | Protein, Serum | 0.05 | 0.03 | Increase |  |
|  | 100 (NR) | 105 (NR) | IgM | Protein, Serum | 0.61 | 0.56 | No change |  |
|  | 100 (NR) | 105 (NR) | IgG | Protein, Serum | 5.22 | 5.39 | No change |  |
| Korany 2023^33^ | 37 (NR) | 30 (NR) | E-cadherin | Protein, Skin | NR | NR | Increase | NR |
| Lai 2021^34^ | 15 (44.3) | 15 (45.4) | IL-4 | Protein, Serum | 2.5 | 1 | Increase | >3 months |
| Machado 2017^35^ | 70 (NR) | 42 (NR) | anti-CMV IgG | Protein, Skin | NR | NR | Increase | NR |
|  | 70 (NR) | 42 (NR) | anti-HSV1 IgG | Protein, Skin | NR | NR | Increase |  |
| Mahmoud 2022^36^ | 27 (35.5) | 30 (35.5) | Aquaporin 3 | Protein, Skin | 0.704 | 1 | Decrease | NR |
| Matsushita 2007^37^ | 20 (NR) | 30 (NR) | BAFF | Protein, Blister Fluid | NR | NR | No change | Untreated |
| Mejri 2011^38^ | 51 (45.3) | 50 (NR) | anti-gliadin IgG | Protein, Serum | NR | NR | Increase | Untreated (21), on treatment (30) |
|  | 51 (45.3) | 50 (NR) | anti-reticulin IgG | Protein, Serum | NR | NR | Increase |  |
|  | 51 (45.3) | 50 (NR) | anti-cardiolipin IgM | Protein, Serum | NR | NR | Increase |  |
| Miguel 2022^39^ | 131 (48) | 57 (59) | anti-Dsg2 | Protein, Skin/Mucosa | 0.18 | 0.102 | Increase | >2 months |
| Miyamoto 2018^40^ | 10 (NR) | 10 (NR) | VEGF | Protein, Skin | 1 | 0.9 | Increase | NR |
| Mortazavi 2014^41^ | 43 (41.1) | 45 (38.1) | IL-1**β** | Protein, Serum | 0.074 | 0.148 | No change | Untreated |
|  | 43 (41.1) | 45 (38.1) | IL-6 | Protein, Serum | 4.1 ± 5.9 | 2.031 ± 2.7 | No change |  |
| Mortazavi 2015^42^ | 82 (44.1) | 131 (42.5) | helicobacter pylori IgG | Protein, Serum | 0.793 | 0.595 | Increased | Untreated |
|  | 82 (44.1) | 131 (42.5) | strongyloides stercoralis IgG | Protein, Serum | 0.695 | 0.16 | Increased |  |
| Nakashima 2007^43^ | 16 (55.7) | 17 (61.7) | CCL11 | Protein, Serum | NR | NR | No change | Untreated |
|  | 16 (55.7) | 17 (61.7) | CXCL1 | Protein, Serum | NR | NR | No change |  |
|  | 16 (55.7) | 17 (61.7) | CXCL10 | Protein, Serum | NR | NR | No change |  |
|  | 16 (55.7) | 17 (61.7) | CCL3 | Protein, Serum | NR | NR | No change |  |
|  | 16 (55.7) | 17 (61.7) | CCL8 | Protein, Serum | NR | NR | No change |  |
|  | 16 (55.7) | 17 (61.7) | CCL7 | Protein, Serum | NR | NR | No change |  |
|  | 16 (55.7) | 17 (61.7) | CCL4 | Protein, Serum | NR | NR | No change |  |
|  | 16 (55.7) | 17 (61.7) | CCL3 | Protein, Serum | NR | NR | No change |  |
|  | 16 (55.7) | 17 (61.7) | CXCL9 | Protein, Serum | NR | NR | No change |  |
|  | 16 (55.7) | 17 (61.7) | CCL5 | Protein, Serum | NR | NR | No change |  |
| Namazi 2010^44^ | 22 (46.8) | 21 (43.2) | MIF | Protein, Serum | 11.99 ± 1.63 | 1.83 ± 0.22 | Increase | >1 month |
| Narbutt 2008^45^ | 19 (NR) | 24 (NR) | IL-6 | Protein, Serum | 3.38 | 1.97 | Increase | Untreated |
| Naseer 2014^46^ | 200 (NR) | 128 (NR) | anti-Dsg1 | Protein, Serum | NR | NR | Increase | NR |
|  | 200 (NR) | 128 (NR) | anti-Dsg3 | Protein, Serum | NR | NR | Increase |  |
| Qian 2014^47^ | 13 (NR) | 7 (NR) | BAFF | Protein, Serum | NR | NR | No change | NR |
| Satyam 2009^48^ | 70 (42.1) | 50 (37.1) | IFN-y | Protein, Serum | 2.9 ± 2.2 | 5.75 ± 3.03 | Decrease | >1 month |
|  | 70 (42.1) | 50 (37.1) | IL-10 | Protein, Serum | 33.15 ± 16.1 | 6.19 ± 1.34 | Increase |  |
|  | 70 (42.1) | 50 (37.1) | IL-2 | Protein, Serum | 2.28 ± 1.5 | 4.24 ± 1.56 | Decrease |  |
|  | 70 (42.1) | 50 (37.1) | IL-4 | Protein, Serum | 12.64 ± 7.9 | 4.38 ± 1.86 | Increase |  |
| Seiffert-Sinha 2015^49^ | NR (NR) | NR (NR) | IL-13 | Protein, Serum | NR | NR | Increase | NR |
| Seiffert-Sinha 2018^50^ | 280 (52.9) | 167 (46) | Anti-Tg | Protein, Serum | 0.068 | 0.006 | Increase | NR |
|  | 280 (52.9) | 167 (46) | Anti-TPO | Protein, Serum | 0.139 | 0.072 | Increase |  |
| Senger 2017^51^ | 87 (52.9) | 49 (50.6) | HSV1 IgG | Protein, Serum | 1.18 ± 0.94 | 0.86 ± 0.72 | Increase | Untreated (12), on treatment (44) |
| Shah 2016^52^ | 21 (55.8) | 25 (59.9) | TAC | Protein, Serum | 278.8 ± 74.3 | 368 ± 101.55 | Decrease | NR |
| Shamsabadi 2015^53^ | 43 (NR) | 40 (NR) | NLRC4 | mRNA, Skin | 3.2 ± 0.26 | 1 ± 0.22 | Increase | Untreated |
|  | 43 (NR) | 40 (NR) | NLRP1 | mRNA, Skin | 4.5 ± 1.48 | 1 ± 0.24 | Increase |  |
| Sharma 2014^54^ | 40 (NR) | 40 (NR) | IFN-y | Protein, Serum | NR | NR | Increase | NR |
|  | 40 (NR) | 40 (NR) | IL-17A | Protein, Serum | NR | NR | Increase |  |
|  | 40 (NR) | 40 (NR) | TGF-β | Protein, Serum | NR | NR | Increase |  |
| Singh 2019^55^ | 30 (NR) | 30 (NR) | IL-10 | Protein, Serum | NR | NR | Decrease | NR |
|  | 30 (NR) | 30 (NR) | IL-17A | Protein, Serum | NR | NR | Increase |  |
|  | 30 (NR) | 30 (NR) | IL-2 | Protein, Serum | NR | NR | Increase |  |
|  | 30 (NR) | 30 (NR) | IL-6 | Protein, Serum | NR | NR | Increase |  |
|  | 30 (NR) | 30 (NR) | TGF-β | Protein, Serum | NR | NR | Decrease |  |
| Sliwiak 2021^56^ | 5 (NR) | 5 (NR) | Prx1 | Protein, Serum | 11897 | 6166 | Increase | NR |
|  | 5 (NR) | 5 (NR) | Prx4 | Protein, Serum | 5555 | 24045 | Decrease |  |
|  | 5 (NR) | 5 (NR) | Trx1 | Protein, Serum | 12220 | 4296 | Increase |  |
| Sobeih 2020^57^ | 25 (45) | 25 (41) | MAPKAPK2 | Protein, Serum | 3.33 ± 1.3 | 1.45 ± 0.68 | Increase | Untreated |
|  | 25 (45) | 25 (41) | P38 MAPK | Protein, Serum | 2227.6 ± 928 | 1780.7 ± 135 | Increase |  |
| Stern 2008^58^ | 15 (57) | 15 (NR) | IFN-y | Protein, Skin | NR | NR | Increase | NR |
|  | 15 (57) | 15 (NR) | IL-6 | Protein, Skin | NR | NR | Increase |  |
|  | 15 (57) | 15 (NR) | IL-8 | Protein, Skin | NR | NR | Increase |  |
|  | 15 (57) | 15 (NR) | MHC II | Protein, Serum | 0.017 | 0.002 | Increase |  |
| Sugiyama 2007^59^ | 11 (58) | 15 (54) | FOXP3 | mRNA, Serum | 3.3 ± 0.3 | 15.5 ± 0.7 | Decrease | Untreated (5), on treatment (6) |
| Sun 2003^60^ | 7 (NR) | 77 (NR) | IL-6 | Protein, Serum | 6.8 ± 7.6 | 2.0 ± 1.5 | Increase | NR |
| Tanita 2017^61^ | NR (NR) | NR (NR) | CXCL5 | Protein, Serum | NR | NR | Increase | NR |
|  | NR (NR) | NR (NR) | CD163 | Protein, Serum | NR | NR | Increase |  |
| Watanabe 2007^62^ | 15 (57.1) | 15 (52.5) | APRIL | Protein, Serum | NR | NR | No change | Untreated |
|  | 10 (55.6) | 12 (53.9) | CD40L | Protein, Serum | NR | NR | No change |  |
| Xu 2020^63^ | 26 (NR) | 20 (NR) | FOXP3 | RNA, Serum | NR | NR | Decrease | NR |
|  | 26 (NR) | 20 (NR) | miR-338-3p | RNA, Serum | 2.5 | 1 | Increase |  |
|  | 26 (NR) | 20 (NR) | RUNX1 | RNA, Serum | 0.75 | 1 | Decrease |  |
| Yavuz 2020^64^ | 30 (45.7) | 30 (46.7) | Pentraxin 3 | Protein, Serum | 3.1 ± 1.5 | 3.1 ± 4.7 | Increase | Untreated |
| Yu 2016^65^ | NR (NR) | NR (NR) | IL-17A | Protein, Serum | 860 ± 314 | 383 ± 121 | Increase | Untreated |
|  | NR (NR) | NR (NR) | TGF-β | Protein, Serum | 0.63 ± 0.078 | 0.34 ± 0.098 | Increase |  |
| Zebrowska 2017^66^ | 23 (58.6) | 20 (61.6) | IL-17A | Protein, Serum | 2.2 ± 0.08 | 0.89 ± 0.12 | Increase | Untreated |
|  | 23 (58.6) | 20 (61.6) | IL-36 | Protein, Serum | 21.55 ± 1.34 | 5.48 ± 3.11 | Increase |  |

1. Abida O, Ben Mansour R, Gargouri B, Ben Ayed M, Masmoudi A, Turki H, et al. Catalase and lipid peroxidation values in serum of tunisian patients with pemphigus vulgaris and foliaceus. Biol Trace Elem Res. 2012;150(1-3):74-80.

2. Alecu M, Ursaciuc C, Surcel M, Coman G, Ciotaru D, Dobre M. CD28 T-cell costimulatory molecule expression in pemphigus vulgaris. Journal of the European Academy of Dermatology and Venereology. 2009;23(3):288-91.

3. Ali S, Kelly C, Challacombe SJ, Donaldson ANA, Bhogal BS, Setterfield JF. Serum and salivary IgG and IgA antibodies to desmoglein 3 in mucosal pemphigus vulgaris. Br J Dermatol. 2016;175(1):113-21.

4. Ameglio F, D'Auria L, Cordiali-Fei P, Mussi A, Valenzano L, D'Agosto G, et al. Bullous pemphigoid and pemphigus vulgaris: Correlated behaviour of serum VEGF, sE-selectin and TNF-alpha levels. J Biol Regul Homeost Agents. 1997;11(4):148-53.

5. Ansari MA, Singh PK, Dar SA, Rai G, Akhter N, Pandhi D, et al. Deregulated phenotype of autoreactive Th17 and Treg clone cells in pemphigus vulgaris after in-vitro treatment with desmoglein antigen (Dsg-3). Immunobiology. 2023;228(2):152340.

6. Asashima N, Fujimoto M, Watanabe R, Nakashima H, Yazawa N, Okochi H, et al. Serum levels of BAFF are increased in bullous pemphigoid but not in pemphigus vulgaris. Br J Dermatol. 2006;155(2):330-6.

7. Asothai R, Anand V, Das D, Antil PS, Khandpur S, Sharma VK, et al. Distinctive Treg associated CCR4-CCL22 expression profile with altered frequency of Th17/Treg cell in the immunopathogenesis of Pemphigus Vulgaris. 2015;220(10):1129-35.

8. Baroni A, De Filippis A, Buommino E, Satriano RA, Cozza V. Osteopontin, a protein with cytokine-like properties: A possible involvement in pemphigus vulgaris. Archives of Dermatological Research. 2012;304(3):237-40.

9. Bhol KC, Rojas AI, Khan IU, Ahmed AR. Presence of interleukin 10 in the serum and blister fluid of patients with pemphigus vulgaris and pemphigoid. Cytokine. 2000;12(7):1076-83.

10. Chen J, Zhang Y, Liang Y, Zhao M, Long H, Xiao R, et al. Regulatory effects of Nr4a2 on Th2 cells from patients with pemphigus vulgaris. Oncotarget. 2018;9(13):11258-67.

11. Christie E, Kozik I, Seiffert K, Sinha A. 043 Deep immunoprofiling in pemphigus reveals significant shifts in dendritic-, natural killer- and T cell compartments at the single-cell level. J Invest Dermatol. 2022;142(8 Supplement):S8.

12. Czech W, Schaller J, Schopf E, Kapp A. Granulocyte activation in bullous diseases: Release of granular proteins in bullous pemphigoid and pemphigus vulgaris. Journal of the American Academy of Dermatology. 1993;29(2 I):210-5.

13. Daneshvar E, Tavakolpour S, Mahmoudi H, Daneshpazhooh M, Teimourpour A, Aslani S, et al. Elevated serum level of B-cell activating factor (BAFF) after rituximab therapy in pemphigus vulgaris patients suggests a possible therapeutic efficacy of B-cell depletion therapies combined with anti-BAFF agents. Int J Dermatol. 2023;62(4):567-74.

14. Das D, Anand V, Khandpur S, Sharma VK, Sharma A. T helper type 1 polarizing gammadelta T cells and Scavenger receptors contribute to the pathogenesis of Pemphigus vulgaris. Immunology. 2018;153(1):97-104.

15. Das D, Singh A, Antil PS, Sharma D, Arava S, Khandpur S, et al. Distorted frequency of dendritic cells and their associated stimulatory and inhibitory markers augment the pathogenesis of pemphigus vulgaris. Immunol Res. 2020;68(6):353-62.

16. Das D, Arava S, Khandpur S, Santosh K, Akhtar S, Sharma A. Dominance and improved survivability of human gammadeltaT17 cell subset aggravates the immunopathogenesis of pemphigus vulgaris. Immunol Res. 2023((Das, Santosh, Akhtar, Sharma) Department of Biochemistry, All India Institute of Medical Sciences, New Delhi 110029, India(Arava) Department of Pathology, All India Institute of Medical Sciences, New Delhi, India(Khandpur) Department of Dermatology & Ven).

17. Luciano DA, Claudio B, Anna M, Giovanna DA, Clara De S, Bernardino G, et al. Cytokines in the sera of patients with pemphigus vulgaris: interleukin-6 and tumour necrosis factor-alpha levels are significantly increased as compared to healthy subjects and correlate with disease activity. Eur Cytokine Netw. 1997;8(4):383-7.

18. Deng Y, Huang S, Xiong X. The imbalance of gut microbiota and its correlation with plasma inflammatory cytokines in pemphigus vulgaris patients. Journal of the Dermatology Nurses' Association. 2020;12(2).

19. Dhandha MM, Seiffert-Sinha K, Sinha AA. Specific immunoglobulin isotypes correlate with disease activity, morphology, duration and HLA association in Pemphigus vulgaris. Autoimmunity. 2012;45(7):516-26.

20. Echigo T, Hasegawa M, Shimada Y, Inaoki M, Takehara K, Sato S. Both Th1 and Th2 chemokines are elevated in sera of patients with autoimmune blistering diseases. Archives of Dermatological Research. 2006;298(1):38-45.

21. El-Eriny A, Genedy R, Swelem R, El-Maghraby EM. Assessment of serum a proliferation-induced ligand level in patients with pemphigus vulgaris. Journal of the Egyptian Women's Dermatologic Society. 2018;15(3):122-6.

22. El-Komy MHM, Samir N, Shaker OG. Estimation of vitamin D levels in patients with pemphigus vulgaris. Journal of the European Academy of Dermatology and Venereology. 2014;28(7):859-63.

23. Ergun EZ, Aoki R, Horvath ON, Hartmann D, Satoh TK, Calabrese L, et al. Divergent in situ expression of IL-31 and IL-31RA between bullous pemphigoid and pemphigus vulgaris. Exp Dermatol. 2023;32(9):1412-9.

24. Ernst N, Schulz A, Friedrich M, Kasperkiewicz M, Gross N, Schmidt E, et al. Expression of the checkpoint receptors PD-1 and Tim-3 is increased in autoimmune blistering diseases. Exp Dermatol. 2019;28(3):e78-e9.

25. Fujimura T, Kakizaki A, Furudate S, Aiba S. A possible interaction between periostin and CD163+ skin-resident macrophages in pemphigus vulgaris and bullous pemphigoid. Exp Dermatol. 2017;26(12):1193-8.

26. Funakoshi T, Lunardon L, Ellebrecht CT, Nagler AR, O'Leary CE, Payne AS. Enrichment of total serum IgG4 in patients with pemphigus. Br J Dermatol. 2012;167(6):1245-53.

27. Gunther C, Carballido-Perrig N, Kopp T, Carballido JM, Pfeiffer C. CCL18 is expressed in patients with bullous pemphigoid and parallels disease course. Br J Dermatol. 2009;160(4):747-55.

28. Hayta SB, Guner R, Akyol M. Blood mean platelet volume may be predictive for disease course in the cases with pemphigus vulgaris. Biomedical Research (India). 2017;28(9):4223-7.

29. Joshi N, Minz RW, Anand S, Parmar NV, Kanwar AJ. Vitamin D deficiency and lower TGF-beta/IL-17 ratio in a North Indian cohort of pemphigus vulgaris. BMC Res Notes. 2014;7((Minz, Anand, Parmar, Kanwar) Department of Immunopathology, Post Graduate Institute of Medical Education and Research, Chandigarh 160012, India. rwminz.minz88@gmail.com):536.

30. Karimi A, Esmaili N, Ranjkesh M, Zolfaghari MA. Expression of human endogenous retroviruses in pemphigus vulgaris patients. Mol Biol Rep. 2019;46(6):6181-6.

31. Ketabi Y, Nasiri S, Kheirodin M, Tavakolpour S, Mozafari N. The elevated level of osteopontin in patients with pemphigus vulgaris: A cytokine-like protein with a therapeutic potential. Dermatol Ther. 2019;32(4):e12973.

32. Khil'chenko S, Boch K, van Beek N, Vorobyev A, Zillikens D, Schmidt E, et al. Alterations of Total Serum Immunoglobulin Concentrations in Pemphigus and Pemphigoid: Selected IgG2 Deficiency in Bullous Pemphigoid. Frontiers in Medicine. 2020;7((Khil'chenko, Schmidt, Ludwig) Lubeck Institute of Experimental Dermatology, University of Lubeck, Lubeck, Germany(Khil'chenko, Boch, van Beek, Vorobyev, Zillikens, Schmidt, Ludwig) Center for Research on Inflammation of the Skin, University of Lubeck, Lu):472.

33. Korany MM, El-Kalioby M, Saleh MA, Youssef RM. 706 The pattern of expression and distribution of E-cadherin in pemphigus vulgaris. J Invest Dermatol. 2023;143(5 Supplement):S122.

34. Lai K, Zhang W, Li S, Zhang Z, Xie S, Xu M, et al. mTOR pathway regulates the differentiation of peripheral blood Th2/Treg cell subsets in patients with pemphigus vulgaris. Acta biochimica et biophysica Sinica. 2021;53(4):438-45.

35. Machado ARDSR, La Serra L, Turatti A, Machado AM, Roselino AM. Herpes simplex virus 1 and cytomegalovirus are associated with pemphigus vulgaris but not with pemphigus foliaceus disease. Exp Dermatol. 2017;26(10):966-8.

36. Mahmoud SB, Saleh MA, Aziz MA, Amer MA. Downregulation of aquaporin 3 in patients with pemphigus vulgaris. Wound Repair Regen. 2022;30(4):448-52.

37. Matsushita T, Hasegawa M, Matsushita Y, Echigo T, Wayaku T, Horikawa M, et al. Elevated serum BAFF levels in patients with localized scleroderma in contrast to other organ-specific autoimmune diseases. Exp Dermatol. 2007;16(2):87-93.

38. Mejri K, Abida O, Kallel-Sellami M, Haddouk S, Laadhar L, Zarraa IR, et al. Spectrum of autoantibodies other than anti-desmoglein in pemphigus patients. Journal of the European Academy of Dermatology and Venereology. 2011;25(7):774-81.

39. Miguel MCB, Julio TA, Vernal S, de Paula NA, Lieber A, Roselino AM. Autoantibodies against desmoglein 2 are not pathogenic in pemphigus. An Bras Dermatol. 2022;97(2):145-56.

40. Miyamoto D, Maruta CW, Santi CG, Zoroquiain P, Dias ABT, Mansure JJ, et al. Exploring the in situ expression of vascular endothelial growth factor and endoglin in pemphigus foliaceus variants and pemphigus vulgaris. Journal of the European Academy of Dermatology and Venereology. 2018;32(11):1954-8.

41. Mortazavi H, Esmaili N, Khezri S, Khamesipour A, Farahani IV, Daneshpazhooh M, et al. The effect of conventional immunosuppressive therapy on cytokine serum levels in pemphigus vulgaris patients. Iranian Journal of Allergy, Asthma and Immunology. 2014;13(3):174-83.

42. Mortazavi H, Hejazi P, Khamesipour A, Mohebali M, Ehsani AH, Mohammadi Y, et al. Frequency of seropositivity against infectious agents amongst pemphigus vulgaris patients: A case-control study on Strongyloides stercoralis, Helicobacter pylori, Toxoplasma gondii, Leishmania major, and Epstein-Barr virus. Int J Dermatol. 2015;54(11):e458-e65.

43. Nakashima H, Fujimoto M, Asashima N, Watanabe R, Kuwano Y, Yazawa N, et al. Serum chemokine profile in patients with bullous pemphigoid. Br J Dermatol. 2007;156(3):454-9.

44. Namazi MR, Fallahzadeh MK, Shaghelani H, Kamali-Sarvestani E. Marked elevation of serum macrophage migration inhibitory factor levels in patients with pemphigus vulgaris. Int J Dermatol. 2010;49(2):146-8.

45. Narbutt J, Lukamowicz J, Bogaczewicz J, Sysa-Jedrzejowska A, Torzecka JD, Lesiak A. Serum concentration of interleukin-6 is increased both in active and remission stages of pemphigus vulgaris. Mediators Inflamm. 2008;2008((Narbutt, Bogaczewicz, Sysa-Jedrzejowska, Lesiak) Department of Dermatology, Medical University of Lodz, 94-017 Lodz, Poland(Lukamowicz) Laboratory of Immunochemical Research, Polish Mother's Memorial Hospital Research Institute, 93-338 Lodz, Poland(Torze):875394.

46. Naseer SY, Seiffert-Sinha K, Sinha Dermatology AA. Anti-desmoglein 1 and -3 profiles as predictors of disease variation in pemphigus vulgaris. J Invest Dermatol. 2014;134(SUPPL. 1):S10.

47. Qian H, Kusuhara M, Li X, Tsuruta D, Tsuchisaka A, Ishii N, et al. B-cell activating factor detected on both naive and memory B cells in bullous pemphigoid. Exp Dermatol. 2014;23(8):596-605.

48. Satyam A, Khandpur S, Sharma VK, Sharma A. Involvement of TH1/TH2 cytokines in the pathogenesis of autoimmune skin diseasepemphigus vulgaris. Immunol Invest. 2009;38(6):498-509.

49. Seiffert-Sinha K, Welch EZ, Dey-Rao R, Sinha AA. IL-13 receptor alpha 1 downregulation as a protective mechanism and therapeutic target in pemphigus. J Invest Dermatol. 2015;135(SUPPL. 1):S12.

50. Seiffert-Sinha K, Khan S, Attwood K, Gerlach JA, Sinha AA. Anti-thyroid peroxidase reactivity is heightened in pemphigus vulgaris and is driven by human leukocyte antigen status and the absence of desmoglein reactivity. Front Immunol. 2018;9(APR):625.

51. Senger P, Abidi N, Lin DM, Seiffert-Sinha K, Sinha AA. Positive correlation of Anti-Herpes simplex type I virus antibody levels with pemphigus vulgaris disease status and activity in a large patient cohort. Eur J Dermatol. 2017;27(2):132-8.

52. Shah AA, Dey-Rao R, Seiffert-Sinha K, Sinha AA. Increased oxidative stress in pemphigus vulgaris is related to disease activity and HLA-association. Autoimmunity. 2016;49(4):248-57.

53. Shamsabadi RM, Basafa S, Yarahmadi R, Goorani S, Khani M, Kamarehei M, et al. Elevated Expression of NLRP1 and IPAF Are Related to Oral Pemphigus Vulgaris Pathogenesis. Inflammation. 2015;38(1):205-8.

54. Sharma V, Tembhre M. T-helper and regulatory T cell cytokines and their correlation with desmoglein antibody levels in pemphigus vulgaris. Journal of the American Academy of Dermatology. 2014;70(5 SUPPL. 1):AB99.

55. Singh PK, Das S, Ansari MA, Dar SA, Rai G. In-vitro functional responses of dsg3-specific autoreactive Treg and Th17 cells in Pemphigus vulgaris. Eur J Immunol. 2019;49(Supplement 3):944.

56. Sliwiak P, Folwarczny E, Didona D, Fink S, Wiegand C, Hanschmann EM, et al. Redox enzymes of the thioredoxin family as potential and novel markers in pemphigus. Oxid Med Cell Longev. 2021;2021((Sliwiak, Folwarczny, Didona, Hertl, Hudemann) Department of Dermatology and Allergology, Philipps University Marburg, Marburg, Germany(Fink, Wiegand) Department of Dermatology, Jena University Medical Center, Jena, Germany(Hanschmann) Department of Neuro):6672693.

57. Sobeih S, Saleh M, Kholoussi S, El Khateeb E, Kadry D. Phosphorylated p38 mitogen-activated kinase in patients with pemphigus vulgaris: A case-controlled study. Journal of the Egyptian Women's Dermatologic Society. 2020;17(1):31-7.

58. Stern JNH, Keskin DB, Barteneva N, Zuniga J, Yunis EJ, Ahmed AR. Possible role of natural killer cells in pemphigus vulgaris - Preliminary observations. Clin Exp Immunol. 2008;152(3):472-81.

59. Sugiyama H, Matsue H, Nagasaka A, Nakamura Y, Tsukamoto K, Shibagaki N, et al. CD4+CD25high regulatory T cells are markedly decreased in blood of patients with pemphigus vulgaris. Dermatology. 2007;214(3):210-20.

60. Sun A, Chia JS, Chang YF, Chiang CP. Levamisole and Chinese medicinal herbs can modulate the serum interleukin-6 level in patients with recurrent aphthous ulcerations. J Oral Pathol Med. 2003;32(4):206-14.

61. Tanita K, Fujimura T, Kakizaki A, Furudate S, Aiba S. Immunomodulatory effects of periostin on CD163+ skin-resident macrophages in pemphigus vulgaris and bullous pemphigoid. J Dermatol Sci. 2017;86(2):e3-e4.

62. Watanabe R, Fujimoto M, Yazawa N, Nakashima H, Asashima N, Kuwano Y, et al. Increased serum levels of a proliferation-inducing ligand in patients with bullous pemphigoid. J Dermatol Sci. 2007;46(1):53-60.

63. Xu M, Liu Q, Li S, Zhang W, Huang X, Han K, et al. Increased expression of miR-338-3p impairs Treg-mediated immunosuppression in pemphigus vulgaris by targeting RUNX1. Exp Dermatol. 2020;29(7):623-9.

64. Yavuz IH, Yavuz GO. The role of pentraxin 3 in pemphigus vulgaris. Postepy Dermatologii i Alergologii. 2020;37(4):503-7.

65. Yu XX, Fu P. Study on Th17/Treg cells and related cytokine IL-17/TGF-beta in pemphigus vulgaris. Journal of Clinical Dermatology. 2016;45(5):332-4.

66. Zebrowska A, Wozniacka A, Juczynska K, Ociepa K, Waszczykowska E, Szymczak I, et al. Correlation between IL36alpha and IL17 and Activity of the Disease in Selected Autoimmune Blistering Diseases. Mediators Inflamm. 2017;2017((Zebrowska, Wozniacka, Juczynska, Ociepa, Waszczykowska) Department of Dermatology and Venereology, Medical University of Lodz, Lodz, Poland(Szymczak, Pawliczak) Department of Immunopathology, Chr. of Allergy, Immunology and Dermatology, Medical Universit):8980534.

**Supplementary Table 2.** T_h_1 biomarkers in patients with PV. Biomarker levels in PV patients vs control; green = increase, yellow = no change, red = decrease.

| **Biomarker** | **Study** | **N patients** | **Weighted association** |
| --- | --- | --- | --- |
| IL-8 | Deng 2020^27^ | 18 | 1 |
|  | Stern 2008^28^ | 15 |  |
| TNF-α | D’auria 1997^16^ | 25 | 1 |
| CXCL9 | Echigo 2006^29^ | 19 | 0.54 |
|  | Nakashima 2007^30^ | 16 |  |
| IL-1**β** | Deng 2020^27^ | 18 | 0.30 |
|  | Mortazavi 2014^31^ | 43 |  |
| IFN-γ | Das 2018^32^ | 30 | 0.24 |
|  | Das 2023^33^ | 30 |  |
|  | Satyam 2009^34^ | 70 |  |
|  | Sharma 2014^35^ | 40 |  |
|  | Stern 2008^28^ | 15 |  |
| CCL3 | Nakashima 2007^30^ | 16 | 0 |
| CXCL10 | Nakashima 2007^30^ | 16 | 0 |
| IL-2 | Satyam 2009^34^ | 70 | -0.40 |
|  | Singh 2019^36^ | 30 |  |

Abbreviations: c-c motif chemokine ligand (CCL), c-x-c motif chemokine ligand (CXCL), interferon (IFN), interleukin (IL) and tumor necrosis factor (TNF).

**Supplementary Table 3.** T_h_2 biomarkers in patients with PV. Biomarker levels in PV patients vs control; green = increase, yellow = no change, red = decrease.

| **Biomarker** | **Study** | **N patients** | **Weighted association** |
| --- | --- | --- | --- |
| IL-13 | Chen 2018^37^ | 19 | 1 |
| IL-4 | Chen 2018^37^ | 19 | 1 |
|  | Das 2018^32^ | 30 |  |
|  | Das 2023^33^ | 30 |  |
|  | Lai 2021^38^ | 15 |  |
|  | Satyam 2009^34^ | 70 |  |
| IL-6 | D’auria 1997^16^ | 25 | 0.69 |
|  | Mortazavi 2014^31^ | 43 |  |
|  | Narbutt 2008^39^ | 19 |  |
|  | Singh 2019^36^ | 30 |  |
|  | Stern 2008^28^ | 15 |  |
|  | Sun 2003^40^ | 7 |  |
| IL-10 | Bhol 2000^41^ | 32 | 0.58 |
|  | Fujimura 2017^42^ | 10 |  |
|  | Satyam 2009^34^ | 70 |  |
|  | Singh 2019^36^ | 30 |  |
| CCL22 | Asothai 2015^43^ | 30 | 0 |
|  | Das 2023^33^ | 30 |  |
| IL-31 | Ergun 2023^44^ | 11 | 0 |
| IL-31RA | Ergun 2023^44^ | 11 | 0 |
| CCL2 | Asothai 2015^43^ | 30 | -0.5 |
|  | Asothai 2015^43^ | 30 |  |

Abbreviations: c-c motif chemokine ligand (CCL) and interleukin (IL).

**Supplementary Table 4.** T_h_17 biomarkers in patients with PV. Biomarker levels in PV patients vs control; green = increase, yellow = no change, red = decrease.

| **Biomarker** | **Study** | **N patients** | **Weighted association** |
| --- | --- | --- | --- |
| CCL20 | Asothai 2015^43^ | 30 | 1 |
|  | Das 2023^33^ | 30 |  |
| CCR6 | Asothai 2015^43^ | 30 | 1 |
|  | Das 2023^33^ | 30 |  |
| IL-17A | Asothai 2015^43^ | 30 | 1 |
|  | Das 2023^33^ | 30 |  |
|  | Joshi 2014^45^ | 30 |  |
|  | Sharma 2014^35^ | 40 |  |
|  | Singh 2019^36^ | 30 |  |
|  | Zebrowska 2017^46^ | 23 |  |
| IL-23 | Das 2023^33^ | 30 | 1 |
| RORγt | Asothai 2015^43^ | 30 | 1 |
|  | Das 2023^33^ | 30 |  |
| IL-6 | D’auria 1997^16^ | 25 | 0.69 |
|  | Mortazavi 2014^31^ | 43 |  |
|  | Narbutt 2008^39^ | 19 |  |
|  | Singh 2019^36^ | 30 |  |
|  | Stern 2008^28^ | 15 |  |
|  | Sun 2003^40^ | 7 |  |
| **IL-1β** | Deng 2020^27^ | 18 | 0.30 |
|  | Mortazavi 2014^31^ | 43 |  |
| TGF-β | Asothai 2015^43^ | 30 | 0.06 |
|  | Das 2023^33^ | 30 |  |
|  | Joshi 2014^45^ | 30 |  |
|  | Sharma 2014^35^ | 40 |  |
|  | Singh 2019^36^ | 30 |  |

Abbreviations: c-c motif chemokine ligand (CCL), c-c chemokine receptor (CCR), interleukin (IL), retinoic acid receptor related orphan receptor (ROR) and transforming growth factor (TGF).

**Supplementary Table 5.** T_reg_ biomarkers in patients with PV. Biomarker levels in PV patients vs control; green = increase, yellow = no change, red = decrease.

| **Biomarker** | **Study** | **N patients** | **Weighted association** |
| --- | --- | --- | --- |
| CCL17 | Echigo 2006^29^ | 19 | 1 |
| IL-2R | Deng 2020^27^ | 18 | 1 |
| IL-10 | Bhol 2000^41^ | 32 | 0.56 |
|  | Fujimura 2017^42^ | 10 |  |
|  | Satyam 2009^34^ | 70 |  |
|  | Singh 2019^36^ | 30 |  |
| CCL22 | Asothai 2015^43^ | 30 | 0.24 |
|  | Das 2023^33^ | 30 |  |
|  | Echigo 2006^29^ | 19 |  |
| TGF-β | Asothai 2015^43^ | 30 | 0.06 |
|  | Das 2023^33^ | 30 |  |
|  | Joshi 2014^45^ | 30 |  |
|  | Sharma 2014^35^ | 40 |  |
|  | Singh 2019^36^ | 30 |  |
| IL-2 | Satyam 2009^34^ | 70 | -0.40 |
|  | Singh 2019^36^ | 30 |  |
| T_reg_ cells | Alecu 2009^47^ | 16 | -0.72 |
|  | Asothai 2015^43^ | 30 |  |
|  | Christie 2022^48^ | 12 |  |
| CCR4 | Asothai 2015^43^ | 30 | -1 |
| FOXP3 | Asothai 2015^43^ | 30 | -1 |
|  | Das 2023^33^ | 30 |  |
|  | Sugiyama 2007^49^ | 11 |  |
|  | Xu 2020^50^ | 26 |  |

Abbreviations: c-c motif chemokine ligand (CCL), c-c chemokine receptor (CCR), forkhead box protein (FOXP), interleukin (IL) and transforming growth factor (TGF).

**Supplementary Table 6.** B-cell biomarkers and antibodies in patients with PV. Biomarker levels in PV patients vs control; green = increase, yellow = no change, red = decrease.

| **Biomarker** | **Study** | **N patients** | **Weighted association** |
| --- | --- | --- | --- |
| **B-cell biomarkers** | | | |
| TNFSF13A | El-Eriny 2018^51^ | 20 | 1 |
| TNFSF13B | Asashima 2006^52^ | 21 | 0.48 |
|  | Daneshvar 2023^53^ | 50 |  |
|  | Matsushita 2007^54^ | 20 |  |
|  | Qian 2014^55^ | 13 |  |
| B-cells | Alecu 2009^47^ | 16 | 0 |
| **Antibodies** | | | |
| Anti-Dsg1 | Das 2020^56^ | 30 | 1 |
|  | Das 2023^33^ | 30 |  |
|  | Naseer 2014^57^ | 200 |  |
| Anti-Dsg2 | Miguel 2022^58^ | 131 | 1 |
| Anti-Dsg3 | Ansari 2023^59^ | 60 | 1 |
|  | Das 2020^56^ | 30 |  |
|  | Das 2023^33^ | 30 |  |
|  | Naseer 2014^57^ | 200 |  |
| Anti-Dsg3 IgG | Ali 2016^60^ | 23 | 1 |
| Anti-Dsg3 IgG1 | Dhandha 2012^61^ | 92 | 1 |
| Anti-Dsg3 IgG4 | Dhandha 2012^61^ | 92 | 1 |
| IgG1 | Khil'chenko 2020^62^ | 100 | 1 |
| IgG4 | Funakoshi 2012^63^ | 48 | 1 |
|  | Khil'chenko 2020^62^ | 100 |  |
| Anti-Dsg3 IgA | Ali 2016^60^ | 23 | 0.20 |
|  | Dhandha 2012^61^ | 92 |  |
| Anti-Dsg3 IgG3 | Dhandha 2012^61^ | 92 | 0 |
| Anti-Dsg3 IgM | Dhandha 2012^61^ | 92 | 0 |
| IgA | Khil'chenko 2020^62^ | 100 | 0 |
| IgE | Czech 1993^64^ | 9 | 0 |
| IgG2 | Khil'chenko 2020^62^ | 100 | 0 |
| IgM | Khil'chenko 2020^62^ | 100 | 0 |
| IgG | Khil'chenko 2020^62^ | 100 | -0.32 |
|  | Funakoshi 2012^63^ | 48 |  |
| Anti-Dsg3 IgG2 | Dhandha 2012^61^ | 92 | -1 |
| IgG3 | Funakoshi 2012^63^ | 48 | -1 |
|  | Khil'chenko 2020^62^ | 100 |  |

Abbreviations: Desmoglein (Dsg) and tumour necrosis factor super family (TNFSF).

**Supplementary Table 7.** Innate immune system biomarkers in patients with PV. Biomarker levels in PV patients vs control; green = increase, yellow = no change, red = decrease.

| **Biomarker** | **Study** | **N patients** | **Weighted association** |
| --- | --- | --- | --- |
| **Dendritic cells** |  |  |  |
| CD40 | Das 2020^56^ | 30 | 1 |
| CD80 | Das 2020^56^ | 30 | 1 |
| LILRB4 | Das 2020^56^ | 30 | 1 |
| PSGL1 | Das 2020^56^ | 30 | 0 |
| **Eosinophils** | | | |
| CCL11 | Nakashima 2007^30^ | 16 | 0 |
| ECP | Czech 1993^64^ | 9 | 0 |
| **Inflammasome** | | | |
| NLRC4 | Shamsabadi 2015^65^ | 43 | 1 |
| NLRP1 | Shamsabadi 2015^65^ | 43 | 1 |
| **Pentraxins** | | | |
| CRP | Hayta 2017^66^ | 43 | 1 |
| Pentraxin 3 | Yavuz 2020^67^ | 30 | 1 |
| **Macrophage and monocytes** | | | |
| CCL17 | Echigo 2006^29^ | 19 | 1 |
| CD163 | Das 2018^32^ | 30 | 1 |
|  | Fujimura 2017^42^ | 10 |  |
| CD36 | Das 2018^32^ | 30 | 1 |
| MIF | Namazi 2010^68^ | 22 | 1 |
| CCL18 | Gunther 2009^69^ | 11 | 0 |
| CCL3 | Nakashima 2007^30^ | 16 | 0 |
| CCL4 | Nakashima 2007^30^ | 16 | 0 |
| CCL7 | Nakashima 2007^30^ | 16 | 0 |
| CCL8 | Nakashima 2007^30^ | 16 | 0 |
| **Natural killer cells** | | | |
| Natural killer cells | Alecu 2009^47^ | 16 | 0 |
| **Neutrophils** | | | |
| CXCL5 | Fujimura 2017^42^ | 10 | 1 |
| Neutrophils | Hayta 2017^66^ | 43 | 1 |
| CCL5 | Nakashima 2007^30^ | 16 | 0 |
| CXCL1 | Nakashima 2007^30^ | 16 | 0 |
| MPO | Czech 1993^64^ | 9 | 0 |
| **Oxidative stress** | | | |
| Catalase activity | Abida 2012^70^ | 36 | 1 |
| Malondialdehyde | Abida 2012^70^ | 36 | 1 |
| Free thiol | Abida 2012^70^ | 36 | 0 |
| Total antioxidant capacity | Shah 2016^71^ | 21 | -1 |

Abbreviations: c-c motif chemokine ligand (CCL), cluster of differentiation (CD), c-reactive protein (CRP), c-x-c motif chemokine ligand (CXCL), eosinophil cationic protein (ECP), leukocyte immunoglobulin-like receptor B (LILRB), macrophage migratory inhibitory factor (MIF), myeloperoxidase (MPO), nucleotide-binding domain leucine-rich repeat family pyrin domain containing (NLR) and P-selectin glycoprotein ligand (PSGL).

References

27. Deng Y, Huang S, Xiong X. The imbalance of gut microbiota and its correlation with plasma inflammatory cytokines in pemphigus vulgaris patients. Journal of the Dermatology Nurses’ Association. 2020;12(2).**[AQ4]**

28. Stern JNH, Keskin DB, Zuniga J, Barteneva N, Yunis EJ, Ahmed AR. Possible role of natural killer cells in pemphigus vulgaris—Preliminary observations. Clin Exp Immunol. 2008;152(3):472-481. doi:10.1111/j.1365-2249.2008.03638.x

29. Echigo T, Hasegawa M, Shimada Y, Inaoki M, Takehara K, Sato S. Both Th1 and Th2 chemokines are elevated in sera of patients with autoimmune blistering diseases. Arch Dermatol Res. 2006;298(1):38-45. doi:10.1007/s00403-006-0661-5

30. Nakashima H, Fujimoto M, Asashima N, et al. Serum chemokine profile in patients with bullous pemphigoid. Br J Dermatol. 2007;156(3):454-459. doi:10.1111/j.1365-2133.2006.07601.x

31. Mortazavi H, Esmaili N, Khezri S, et al. The effect of conventional immunosuppressive therapy on cytokine serum levels in pemphigus vulgaris patients. Iran J Allergy Asthma Immunol. 2014;13(3):174-183.

32. Das D, Anand V, Khandpur S, Sharma VK, Sharma A. T helper type 1 polarizing gammadelta T cells and Scavenger receptors contribute to the pathogenesis of Pemphigus vulgaris. Immunology. 2018;153(1):97-104. doi:10.1111/imm.12814

33. Das D, Arava S, Khandpur S, Santosh K, Akhtar S, Sharma A. Dominance and improved survivability of human gammadeltaT17 cell subset aggravates the immunopathogenesis of pemphigus vulgaris. Immunol Res. 2023; 72(1): 72-81. doi:10.1007/s12026-023-09413-0

34. Satyam A, Khandpur S, Sharma VK, Sharma A. Involvement of TH1/TH2 cytokines in the pathogenesis of autoimmune skin diseasepemphigus vulgaris. Immunol Invest. 2009;38(6):498-509. doi:10.1080/08820130902943097

35. Sharma V, Tembhre M. T-helper and regulatory T cell cytokines and their correlation with desmoglein antibody levels in pemphigus vulgaris. J Am Acad Dermatol. 2014;70(5 SUPPL. 1):AB99. doi:10.1016/j.jaad.2014.01.410

36. Singh PK, Das S, Ansari MA, Dar SA, Rai G. In-vitro functional responses of dsg3-specific autoreactive Treg and Th17 cells in Pemphigus vulgaris. Eur J Immunol. 2019;49(Supplement 3):944. doi:10.1002/eji.201970400

37. Chen J, Zhang Y, Liang Y, et al. Regulatory effects of Nr4a2 on Th2 cells from patients with pemphigus vulgaris. Oncotarget. 2018;9(13):11258-11267. doi:10.18632/oncotarget.24371

38. Lai K, Zhang W, Li S, et al. MTOR pathway regulates the differentiation of peripheral blood Th2/Treg cell subsets in patients with pemphigus vulgaris. Acta biochimica et biophysica Sinica. 2021;53(4):438-445. doi:10.1093/abbs/gmab008

39. Narbutt J, Lukamowicz J, Bogaczewicz J, Sysa-Jedrzejowska A, Torzecka JD, Lesiak A. Serum concentration of interleukin-6 is increased both in active and remission stages of pemphigus vulgaris. Mediators Inflamm. 2008;2008:875394. doi:10.1155/2008/875394

40. Sun A, Chia JS, Chang YF, Chiang CP. Levamisole and Chinese medicinal herbs can modulate the serum interleukin-6 level in patients with recurrent aphthous ulcerations. J Oral Pathol Med. 2003;32(4):206-214. doi:10.1034/j.1600-0714.2003.00096.x

41. Bhol KC, Rojas AI, Khan IU, Ahmed AR. Presence of interleukin 10 in the serum and blister fluid of patients with pemphigus vulgaris and pemphigoid. Cytokine. 2000;12(7):1076-1083. doi:10.1006/cyto.1999.0642

42. Fujimura T, Kakizaki A, Furudate S, Aiba S. A possible interaction between periostin and CD163+ skin-resident macrophages in pemphigus vulgaris and bullous pemphigoid. Exp Dermatol. 2017;26(12):1193-1198. doi:10.1111/exd.13157

43. Asothai R, Anand V, Das D, et al. Distinctive Treg associated CCR4-CCL22 expression profile with altered frequency of Th17/Treg cell in the immunopathogenesis of Pemphigus Vulgaris. 2015;220(10):1129-1135. doi:10.1016/j.Imbio.2015.06.008

44. Ergun EZ, Aoki R, Horvath ON, et al. Divergent in situ expression of IL-31 and IL-31RA between bullous pemphigoid and pemphigus vulgaris. Exp Dermatol. 2023;32(9):1412-1419. doi:10.1111/exd.14842

45. Joshi N, Minz RW, Anand S, Parmar NV, Kanwar AJ. Vitamin D deficiency and lower TGF-beta/IL-17 ratio in a North Indian cohort of pemphigus vulgaris. BMC Res Notes. 2014;7:536. doi:10.1186/1756-0500-7-536

46. Zebrowska A, Wozniacka A, Juczynska K, et al. Correlation between IL36alpha and IL17 and Activity of the Disease in Selected Autoimmune Blistering Diseases. Mediators Inflamm. 2017;2017:8980534. doi:10.1155/2017/8980534

47. Alecu M, Ursaciuc C, Surcel M, Coman G, Ciotaru D, Dobre M. CD28 T-cell costimulatory molecule expression in pemphigus vulgaris. J Eur Acad Dermatol Venereol. 2009;23(3):288-291. doi:10.1111/j.1468-3083.2008.03035.x

48. Christie E, Kozik I, Seiffert K, Sinha A. 043 Deep immunoprofiling in pemphigus reveals significant shifts in dendritic-, natural killer- and T cell compartments at the single-cell level. J Invest Dermatol. 2022;142(8 Supplement):S8. doi:10.1016/j.jid.2022.05.097

49. Sugiyama H, Matsue H, Nagasaka A, et al. CD4+CD25high regulatory T cells are markedly decreased in blood of patients with pemphigus vulgaris. Dermatology. 2007;214(3):210-220. doi:10.1159/000099585

50. Xu M, Liu Q, Li S, et al. Increased expression of miR-338-3p impairs Treg-mediated immunosuppression in pemphigus vulgaris by targeting RUNX1. Exp Dermatol. 2020;29(7):623-629. doi:10.1111/exd.14111

51. El-Eriny A, Genedy R, Swelem R, El-Maghraby EM. Assessment of serum a proliferation-induced ligand level in patients with pemphigus vulgaris. J Egypt Women’s Dermatol Soc. 2018;15(3):122-126. doi:10.1097/01.EWX.0000542472.35180.f9

52. Asashima N, Fujimoto M, Watanabe R, et al. Serum levels of BAFF are increased in bullous pemphigoid but not in pemphigus vulgaris. Br J Dermatol. 2006;155(2):330-336. doi:10.1111/j.1365-2133.2006.07305.x

53. Daneshvar E, Tavakolpour S, Mahmoudi H, et al. Elevated serum level of B-cell activating factor (BAFF) after rituximab therapy in pemphigus vulgaris patients suggests a possible therapeutic efficacy of B-cell depletion therapies combined with anti-BAFF agents. Int J Dermatol. 2023;62(4):567-574. doi:10.1111/ijd.16363

54. Matsushita T, Hasegawa M, Matsushita Y, et al. Elevated serum BAFF levels in patients with localized scleroderma in contrast to other organ-specific autoimmune diseases. Exp Dermatol. 2007;16(2):87-93. doi:10.1111/j.1600-0625.2006.00485.x

55. Qian H, Kusuhara M, Li X, et al. B-cell activating factor detected on both naive and memory B cells in bullous pemphigoid. Exp Dermatol. 2014;23(8):596-605. doi:10.1111/exd.12421

56. Das D, Singh A, Antil PS, et al. Distorted frequency of dendritic cells and their associated stimulatory and inhibitory markers augment the pathogenesis of pemphigus vulgaris. Immunol Res. 2020;68(6):353-362. doi:10.1007/s12026-020-09166-0

57. Naseer SY, Seiffert-Sinha K, Sinha Dermatology AA. Anti-desmoglein 1 and -3 profiles as predictors of disease variation in pemphigus vulgaris. J Invest Dermatol. 2014;134(SUPPL. 1):S10. doi:10.1038/jid.2014.102

58. Miguel MCB, Julio TA, Vernal S, de Paula NA, Lieber A, Roselino AM. Autoantibodies against desmoglein 2 are not pathogenic in pemphigus. An Bras Dermatol. 2022;97(2):145-156. doi:10.1016/j.abd.2021.06.004

59. Ansari MA, Singh PK, Dar SA, et al. Deregulated phenotype of autoreactive Th17 and Treg clone cells in pemphigus vulgaris after in-vitro treatment with desmoglein antigen (Dsg-3). Immunobiology. 2023;228(2):152340. doi:10.1016/j.imbio.2023.152340

60. Ali S, Kelly C, Challacombe SJ, Donaldson ANA, Bhogal BS, Setterfield JF. Serum and salivary IgG and IgA antibodies to desmoglein 3 in mucosal pemphigus vulgaris. Br J Dermatol. 2016;175(1):113-121. doi:10.1111/bjd.14410

61. Dhandha MM, Seiffert-Sinha K, Sinha AA. Specific immunoglobulin isotypes correlate with disease activity, morphology, duration and HLA association in Pemphigus vulgaris. Autoimmunity. 2012;45(7):516-526. doi:10.3109/08916934.2012.702811

62. Khil’chenko S, Boch K, van Beek N, et al. Alterations of total serum immunoglobulin concentrations in pemphigus and pemphigoid: selected IgG2 deficiency in bullous pemphigoid. Front Med. 2020;7:472. doi:10.3389/fmed.2020.00472

63. Funakoshi T, Lunardon L, Ellebrecht CT, Nagler AR, O’Leary CE, Payne AS. Enrichment of total serum IgG4 in patients with pemphigus. Br J Dermatol. 2012;167(6):1245-1253. doi:10.1111/j.1365-2133.2012.11144.x

64. Czech W, Schaller J, Schopf E, Kapp A. Granulocyte activation in bullous diseases: release of granular proteins in bullous pemphigoid and pemphigus vulgaris. J Am Acad Dermatol. 1993;29(2 I):210-215.

65. Shamsabadi RM, Basafa S, Yarahmadi R, et al. Elevated expression of NLRP1 and IPAF are related to oral pemphigus vulgaris pathogenesis. Inflammation. 2015;38(1):205-208. doi:10.1007/s10753-014-0023-y

66. Hayta SB, Guner R, Akyol M. Blood mean platelet volume may be predictive for disease course in the cases with pemphigus vulgaris. Biomed Res (India). 2017;28(9):4223-4227.

67. Yavuz IH, Yavuz GO. The role of pentraxin 3 in pemphigus vulgaris. Postepy Dermatologii i Alergologii. 2020;37(4):503-507. doi:10.5114/ada.2019.81234

68. Namazi MR, Fallahzadeh MK, Shaghelani H, Kamali-Sarvestani E. Marked elevation of serum macrophage migration inhibitory factor levels in patients with pemphigus vulgaris. Int J Dermatol. 2010;49(2):146-148. doi:10.1111/j.1365-4632.2009.04238.x

69. Gunther C, Carballido-Perrig N, Kopp T, Carballido JM, Pfeiffer C. CCL18 is expressed in patients with bullous pemphigoid and parallels disease course. Br J Dermatol. 2009;160(4):747-755. doi:10.1111/j.1365-2133.2008.08979.x

70. Abida O, Ben Mansour R, Gargouri B, et al. Catalase and lipid peroxidation values in serum of tunisian patients with pemphigus vulgaris and foliaceus. Biol Trace Elem Res. 2012;150(1-3):74-80. doi:10.1007/s12011-012-9497-3

71. Shah AA, Dey-Rao R, Seiffert-Sinha K, Sinha AA. Increased oxidative stress in pemphigus vulgaris is related to disease activity and HLA-association. Autoimmunity. 2016;49(4):248-257. doi:10.3109/08916934.2016.1145675
